# Evaluating implant properties on the implant stability of immediately loaded implants

**DOI:** 10.6026/973206300213296

**Published:** 2025-09-30

**Authors:** Md Kafeel Ahmed, Harinder Singh Mangat, Ananya Bhargava, Monika Tanwar, Mugur Basavanna Jayaprakash, Satyabrata Das, Miral Mehta, Dhirendra Kumar Singh

**Affiliations:** 1 Department of Periodontology, MNR Dental College and Hospital, Sangareddy, Hyderabad Telangana, India; 2 Department of Dental Surgery, General Dentist (India)/Registered Dental Assistant level 2, 506-280 Williamstown Close NW, Airdrie, Alberta, T4B 4B6, Canada; 3 Consultant Orthodontist, Avanti Hospital, Ujjain, Madhya Pradesh, India; 4 Department of Oral and Maxillofacial surgery, SGT University, Gurgaon, Haryana, India; 5 Department of Prosthodontics, Dental Institute, RIMS, Ranchi, Jharkhand, India; 6 Medical Officer (Dental Surgeon), CHC Dasarathpur, Jajpur, Odisha, India; 7 Department of Pediatric and Preventive Dentistry, Karnavati School of Dentistry, Karnavati University, Gandhinagar, Gujarat, India; 8 Department of Periodontology, Kalinga Institute of Dental Sciences, KIIT Deemed to be University, Bhubaneswar, Odisha, India

**Keywords:** Immediate loading, implant stability quotient, surface roughness, implant diameter, resonance frequency analysis, primary stability, dental implants

## Abstract

Immediate implantation of dental implants provides a shortened period of treatment at the cost of high stability requirements. Therefore, it is of interest to assess the impact of implant diameter, length and surface texture on the implant stability quotient (ISQ) of 40 patients on fibroblastic posterior mandibular implants. Primary and secondary ISQ values were much higher in the rough surfaces and wider diameters (p < 0.05), whereas the length of the implants did not have much influence. There was an improvement in ISQ in all groups after 12 weeks. Thus, the success of immediate loading depends on surface roughness and diameter.

## Background:

The dental arena has also shown promise with immediate loading of dental implants being a treatment process with great reliability, efficiency and its cost-effective nature being an added advantage to this treatment process, since it has been shown to decrease the time duration of the treatment process and provide increased patient satisfaction levels. This means of action is done by inserting prosthesis between 48 to 72 hours of placing implants, doing away with the longstanding healing process that takes place by the use of bones when using osseointegration in the conventional method [1]. The main principle behind the success of immediate loading is the degree of the development of the initial stability, also called the mechanical contact between the implant and the bone in situ [2]. Several factors, such as bone type, the design of implants, surgical procedure and the insertion torque, determine the primary implant stability [3]. These include implant-specific features like surface texture, diameter and length and thread pattern, which are essential to provide an increased contact between bone to implants and early osseointegration [4]. Textured surfaces, especially the roughened ones, have been found to enhance the speed as well as volume of bone incorporation when compared to a machined surface, hence adding to higher initial stability [5]. No doubt, resonance frequency analysis (RFA) is a highly popular, non-invasive means of evaluating implant stability that is commonly applied to measure the viability of immediate loading [6]. RFA used to calculate the implant stability quotient (ISQ) gives an objective estimation of primary and secondary stability as a time process [7]. It has been reported in other studies that increasing values of ISQ are associated with better clinical outcomes and fewer incidences of implant failures in the case of immediate loading [8, 9]. Primary stability is considered particularly important in immediate loading procedures because damaged micromotion at the early stages of loading imposes a threat to osseointegration, causing fibrous tissue instead of bone-like tissue to cover around the implant surface. Implants inserted into thick cortical bone appear to achieve a good primary stability as compared to the same insertion of thick cancellous bone because of greater mechanical interlocking [3]. Consequently, there are changes in the design of the implants, including a tapered body and a progressive thread that improve initial fixation in bone of different qualities [4]. Such geometrical adjustments enable a controlled compression of the bone during insertion, which helps in the increased anchoring of the bone mechanically. The surface properties of implants have changed significantly and are now oriented at producing the desired effect, like activating osteoblasts and promoting the osseointegration process, changing smooth (machined) surfaces to any combination of moderately rough or nanostructured ones. To buff the implant surfaces, methods such as sandblasting and acid-etching give motifs of rough surface that will deliver more surface area and enhanced bone-to-implant connection [6]. These surfaces have shown enhanced early bone healing and greater stability in both animal models and clinical trials, which are beneficial to immediate loading. A surface with a moderately rough (Sa 1.0-2.0 mm) finish is no longer debated as the best compromise between biological integration and over-retention of plaque [8].

Biomechanical distribution and initial stability are also dependent on the length and diameter of implants, particularly in areas with low-quality bones, like the posterior maxilla. Increased implant lengths bring with them an increment in bone-to-implant contact area, thus possessing improved stress distribution and wider implants give rise to increased resistance to bending and torsion stresses [1]. They are, however, limited by clinical factors like anatomical factors and bone resorption that tend to determine the size of implants. As such, the maximization of the implant design is imperative when the implants need to be narrow or shorter [9]. In recent studies, it was discovered that the shorter implants, too, can obtain high levels of ISQ values when coupled with better macro- and micro-geometry [2]. The tool of resonance frequency analysis (RFA) offers a reliable and non-injurious way through which clinicians could measure an optimum value of implant stability over time. The ISQ scale is direct, 1 to 100, with a value of above 60 usually regarded as acceptable to immediately load. The factors that may affect ISQ reading include transducer placement, geometry of implants in the body and the density of the surrounding bone. Follow-up monitoring of ISQ values may assist in the assessment of implants so that early signs of progressive instability will also help clinicians make decisions about the prosthetic loading protocols [7]. Therefore, RFA is an advisable supplement to clinical decision-making in the treatment of implants. Even though there are many studies on the stability of implants, there is a scarcity on whether various implant characteristics, e.g., surface topography and geometry, have distinct influences on the stability of immediately loaded implants. Therefore, it is of interest to evaluate implant properties on the implant stability of immediately loaded implants.

## Materials and Methods:

This prospective clinical study was conducted in the Department of Prosthodontics and Implantology after obtaining institutional ethical clearance and written informed consent from all participants. A total of 40 systemically healthy patients, aged between 28 and 62 years, requiring single-tooth implants in the posterior mandible were selected. The inclusion criteria were good oral hygiene, sufficient bone volume for implant placement without the need for grafting and willingness to follow up for the entire study period. Patients with uncontrolled systemic conditions, parafunctional habits, or insufficient bone support were excluded. All implants used in this study were titanium, root-form, endosseous implants with varying design features. Based on the implant characteristics, participants were divided into subgroups according to implant diameter (3.5 mm vs 4.5 mm), length (10 mm vs 12 mm) and surface topography (machined vs roughened surface). All implants were placed following a standardized two-stage surgical protocol under local anesthesia. The osteotomy was prepared using sequential drills recommended by the implant manufacturer and implants were inserted with a torque value not less than 35 Ncm to ensure initial stability. Immediately following implant placement, a transepithelial healing abutment was attached and provisional prostheses were delivered within 48 hours, ensuring minimal occlusal loading. Resonance frequency analysis (RFA) was performed using an Osstell™ device to measure the Implant Stability Quotient (ISQ) at the time of placement (baseline) and after 12 weeks. The RFA probe was positioned perpendicular to the implant and the ISQ value was recorded from two directions (buccolingual and mesiodistal), with the mean value taken for analysis. Data were recorded and tabulated using Microsoft Excel. Statistical analysis was carried out using SPSS version 25.0 (IBM Corp., Armonk, NY, USA). Descriptive statistics such as mean and standard deviation were calculated for ISQ values. One-way ANOVA was used to compare implant stability among different groups based on implant characteristics and paired t-tests were used to assess changes between baseline and 12-week ISQ values. A p-value less than 0.05 were considered statistically significant.

## Results:

A total of 40 implants were placed in 40 patients (22 males, 18 females; mean age: 45.2 ± 8.7 years). All implants achieved primary stability and were successfully loaded within 48 hours. No implant failures or major complications were observed during the 12-week follow-up period. The primary Implant Stability Quotient (ISQ) values recorded at the time of placement varied significantly based on implant surface characteristics, diameter and length. Implants with a roughened surface demonstrated higher mean ISQ values (68.4 ± 2.9) compared to machined surfaces (62.7 ± 3.1), showing a statistically significant difference (p = 0.002). Similarly, implants with a diameter of 4.5 mm exhibited greater primary stability (70.1 ± 3.0) than those with a 3.5 mm diameter (64.2 ± 2.8; p = 0.001). The difference in ISQ values between 10 mm and 12 mm long implants was not statistically significant (p = 0.08), although 12 mm implants showed a marginally higher stability (Table 1 - see PDF). After 12 weeks, all implant groups showed an increase in ISQ values. The highest secondary ISQ was observed in rough-surfaced, 4.5 mm diameter implants (74.6 ± 2.5), while machined-surface, 3.5 mm implants demonstrated lower secondary ISQ (68.3 ± 3.2). The improvement in ISQ from baseline to 12 weeks was statistically significant across all subgroups (p< 0.05), indicating successful osseointegration and stability of immediately loaded implants (Table 2 - see PDF). These findings indicate that implant surface texture and diameter have a notable impact on both initial and long-term stability of immediately loaded implants (Tables 1 - see PDF and 2 - see PDF).

## Discussion:

The study evaluated the effect of the implant features typified as surface texture, implant diameter and implant length, as well as the stability of immediately loaded implants, because on assessed resonance frequency analysis (RFA) as ISQ values. The results demonstrated that the surface roughness, as well as the increased diameter of implants, contributed to both the primary and secondary stability quite a lot, whereas implant length produced a minor but visible effect. These findings are consistent with the existing literature, suggesting that the geometry of implants and the pattern of the implant surface play a central role in ensuring good results related to the immediate loading. It was a very strong impact of surface roughness on the values of both ISQ at baseline and at 12-week follow-up. Implants with rough surfaces were always stabilised more than machined-surface implants, which confirms the literature results of the more bone-to-implant contact and delivery of better osseointegration on medium-rough implants [3]. The surface modifications that enhance the osteoblastic activity were found to produce quick and more permanent compaction of the bone around the implant via sandblasting and acid-etching with animal and human trials [2]. Also, somewhat smooth surfaces (Sa 12m) balance biological growth and resistance to plaque and are beneficial to provide peri-implant health [5]. The stability was also greatly influenced by the diameter of the implants. Both the primary and secondary measurements show that wider implants (4.5 mm) recorded higher ISQ values than narrower implants (3.5 mm). The result is in agreement with previous works that indicate greater implant diameter contributes to better primary stability as a greater bone-to-implant interfacial of ostasis and increased occlusal distribution of the load are attained [8]. Other advantages are also achieved through larger-diameter implants, which provide higher resistance to micromotion, which is an important element of success in immediate loading. Nevertheless, clinicians should also take into consideration the danger of possible bone resorption, which implies that very wide implants can be placed in those regions where the alveolar ridge cannot be widened [6]. Implant length trended positively and was non-statistically significant according to the higher ISQ values, where 12 mm implants performed slightly better than 10 mm implants. The fact is that the longer the implant, the greater the surface area to be in contact with the bone, but the benefit of length may be lost in dense cortical bone where the quality of bone as a factor in stability is more important than length alone [9, 10]. Short implants (8mm) have, in fact and according to some authors, shown even (modest) comparable results when inserted in a favorable bone situation and with a proper loading protocol [11, 12]. Therefore, length would not be as important in attaining stability as implant design and surface property in cases of anatomically challenging conditions.

RFA was found to be a useful method in checking the stability of implants over time. The likelihood of progressive osseointegration and no fibrous encapsulation is shown by the increase in the ISQ values in all groups. An ISQ threshold of 60 has been noted as an acceptable value in immediate loading and the prognosis is proportional to the values beyond 60 [13, 14]. The reliability of measuring ISQ in this study holds at both time points, which makes it an effective diagnostic tool in measuring the amount of implant stability. The results of this research are congruent with various accounts that were published before. According to the study conducted by Degidi *et al.* mean ISQ values above 65 at the time of immediate loading and above 70 after healing were reported with roughened-surface Orthopaedic Replacement [15]. Equally, Pérez-Pevida *et al.* and Souza *et al.* studies highlighted the significance of the initial mechanical participation and alteration surface about the underpinning of the early loading protocols [16, 17]. Results in other clinical studies have confirmed that immediate loading success in implant cases mainly depends on primary stability, which is affected by an implant design and surgery technique [18, 19]. Although this study provides valuable insights, it is not without limitations. The sample size was relatively small and all implants were placed in the posterior mandible, limiting the generalizability of findings to other regions such as the maxilla. Further multicenter studies with larger cohorts and longer follow-up durations are warranted to confirm these results and evaluate long-term outcomes.

## Conclusion:

Implant surface texture and diameter significantly affect the stability of immediately loaded implants, as measured using ISQ.
